# Velocity and Angle Tracking of Fast Targets Using a Bandwidth-Coded Hybrid Chirp FMCW Radar

**DOI:** 10.3390/s26061751

**Published:** 2026-03-10

**Authors:** Burak Gökdemir, Yaser Dalveren, Ali Kara, Mohammad Derawi

**Affiliations:** 1 TUBITAK SAGE, 06484 Ankara, Turkey; burak.gokdemir@tubitak.gov.tr; 2 Graduate School of Natural and Applied Sciences, Gazi University, 06570 Ankara, Turkey; 3 Department of Electrical and Electronics Engineering, Izmir Bakircay University, 35665 Izmir, Turkey; yaser.dalveren@bakircay.edu.tr; 4 Department of Electrical and Electronics Engineering, Faculty of Engineering, Gazi University, 06570 Ankara, Turkey; akara@gazi.edu.tr; 5 Department of Electronic Systems, Norwegian University of Science and Technology, 2815 Gjovik, Norway

**Keywords:** FMCW radar, velocity estimation, unscented Kalman filter, high-velocity target tracking, monopulse angle estimation

## Abstract

**Highlights:**

**What are the main findings?**

- A hybrid FMCW chirp waveform with bandwidth coding enables accurate velocity estimation using only two chirps while improving the maximum unambiguous velocity limitation.
- Unscented Kalman filtering provides more robust and faster convergence than extended Kalman filtering for tracking high-speed targets under nonlinear measurement conditions.

**What are the implications of the main findings?**

- The proposed approach significantly reduces the IF bandwidth and required ADC sampling rate in fast-target scenarios compared to conventional 2D-FFT-based FMCW radar methods.
- The framework is well suited for radar systems requiring reliable tracking of fast-moving targets at short and medium ranges, particularly in single-target applications.

**Abstract:**

Frequency-modulated continuous-wave (FMCW) radars are widely used for range and velocity estimation. However, conventional velocity measurement techniques based on 2D-FFT processing require a large number of chirps and suffer from a maximum unambiguous velocity limitation, which restricts their applicability to high-speed targets. This study addresses these challenges by proposing a hybrid FMCW chirp waveform that employs bandwidth variation between consecutive chirps while maintaining a constant chirp duration. The proposed method enables separation of range- and Doppler-dependent frequency components using only two chirps; thus, it improves the maximum velocity constraint by keeping intermediate-frequency bandwidth and sampling requirements low. In addition, spatial angle estimation is performed using an amplitude-comparison monopulse antenna configuration, allowing single-snapshot angle measurement with low computational complexity. To enhance measurement robustness, extended and unscented Kalman filters are integrated for target tracking. Simulation results demonstrate that the proposed waveform achieves accurate velocity estimation for very high-speed targets and that the unscented Kalman filter consistently outperforms the extended Kalman filter in terms of convergence speed and robustness, particularly under poor initialization and strong nonlinearities. The results confirm that the proposed framework provides an efficient solution for tracking a single, fast-moving, isolated target in a homogeneous environment using FMCW radar systems at short and medium ranges.

## 1. Introduction

Frequency-modulated continuous-wave (FMCW) radars are widely employed in a broad range of applications, including modern surveillance systems, automotive and aviation platforms, and various civilian applications, owing to their low power consumption, high range resolution, and capability for continuous operation. Moreover, their inherently low peak-to-average power ratio results in a low probability of detection, making FMCW radars particularly attractive for covert sensing applications. Compared to conventional pulsed radars, FMCW radars offer several advantages, such as lower average transmit power, shorter measurement intervals, improved resolution at short ranges, and a significantly reduced blind range due to the simultaneous operation of the transmitter and receiver [1,2,3,4]. These characteristics are especially critical in fast-target scenarios, where short update times, minimal blind zones, and accurate close-range measurements are essential for reliable target tracking.

In FMCW radar systems, target range and radial velocity are extracted from the beat signal obtained by mixing the transmitted chirp signal with a delayed version of itself reflected from the target. The resulting intermediate-frequency (IF) signal inherently contains both range-dependent frequency components and Doppler shifts induced by target motion [2]. Joint range–velocity estimation is commonly performed using the two-dimensional fast Fourier transform (2D-FFT) method. For example, a 2D-FFT-based approach using sawtooth waveforms was proposed in [5], where a large number of chirps were required to achieve sufficient velocity resolution. In [6], the maximum unambiguous velocity limitation was addressed by reducing the chirp period. Hybrid waveform strategies have also been explored. For instance, the hybrid chirp technique presented in [7] demonstrated improved robustness against RF spoofing by distinguishing real targets from deceptive signals.

Although range and velocity information are fundamental, they are insufficient for complete spatial separation of targets. Consequently, direction-of-arrival (DOA) estimation techniques have been extensively studied to determine target angular position [8,9]. High-resolution subspace-based methods, such as multiple signal classification (MUSIC) and estimation of signal parameters via rotational invariance techniques (ESPRIT), provide excellent angular accuracy [10,11]. However, these approaches typically require multiple snapshots and incur high computational costs, which limit their applicability in real-time radar systems.

To address these limitations, monopulse-based DOA estimation has been proposed as a computationally efficient alternative, particularly for single-target scenarios. Several studies have evaluated monopulse and other DOA estimators under single-snapshot conditions [8,12]. While monopulse estimation generally exhibits lower angular resolution than multi-snapshot MUSIC-based methods [13], this loss can be largely compensated through target tracking algorithms [8]. Moreover, the choice of DOA estimation technique strongly depends on the operational scenario. When resolving closely spaced targets is required, MUSIC-based methods are preferable [14], whereas in single-target tracking applications, monopulse methods offer advantages in terms of robustness, computational efficiency, and superior performance in low signal-to-noise ratio (SNR) environments [15].

Radar measurements are inevitably affected by sensor noise, environmental disturbances, and target maneuvering, rendering raw measurements unreliable. To mitigate these effects, state estimation and tracking algorithms are employed. Both linear and nonlinear filtering techniques have been investigated for radar tracking applications, and a comprehensive comparison of nonlinear filters is provided in [16]. When the target motion model accurately represents the true dynamics, the Kalman filter can significantly reduce the mean square error between predicted and actual target trajectories. Previous studies have shown that the unscented Kalman filter (UKF) generally outperforms the extended Kalman filter (EKF) in navigation and radar tracking applications, albeit at the cost of increased computational complexity [17,18]. In particular, UKF has demonstrated superior convergence behavior compared to EKF when initial state estimates are poor [19].

Despite the extensive literature on FMCW radar signal processing, several important challenges remain. Conventional velocity estimation approaches based on the 2D-FFT framework require a large number of chirps to achieve adequate velocity resolution and are fundamentally limited by maximum unambiguous velocity constraints. Although reducing the chirp duration can alleviate this limitation, it increases system complexity by requiring wider analog front-end bandwidths and higher analog-to-digital converter (ADC) sampling rates, which in turn degrade noise performance. As a result, achieving high-resolution velocity estimation within short observation intervals remains a critical challenge for fast-moving targets. Furthermore, while monopulse DOA estimation has been well studied theoretically, practical antenna array design and coefficient extraction under 3-D electromagnetic simulation results have not been sufficiently addressed. In addition, existing studies often treat velocity estimation, angle measurement, and target tracking as separate problems, and an integrated framework that jointly considers these aspects is still lacking.

Motivated by these limitations, this paper presents an integrated FMCW radar processing framework for the estimation and tracking of range, velocity, and spatial angle of short- and medium-range fast single targets. A hybrid chirp waveform with constant duration and varying sweep bandwidth is proposed for velocity estimation. By exploiting the resulting separation of IF frequency components in the spectrum, accurate velocity estimation is achieved using only two chirps, enabling high velocity resolution within a short measurement time while improving maximum unambiguous velocity limitation by keeping ADC bandwidth and system noise low. In addition, a practical four-subarray antenna array is designed using HFSS for monopulse-based angle estimation, and the corresponding coefficients are obtained using antenna pattern behavior. Finally, EKF and UKF algorithms are applied to track the measured parameters, and their performance is comparatively evaluated, demonstrating the superior accuracy and robustness of UKF under realistic noise and maneuvering conditions.

The remainder of this paper is organized as follows. Section 2 presents the proposed hybrid chirp waveform and the associated detection and measurement algorithms for range and velocity estimation. Section 3 describes the monopulse angle estimation method and the antenna array design, along with the electromagnetic simulation results used to derive the monopulse coefficients. Section 4 introduces the EKF and UKF algorithms employed for optimal target tracking. Simulation results are presented in Section 5, and the findings are discussed in Section 6. The conclusions are drawn in Section 7.

## 2. FMCW Radar Design

**Figure 1:**
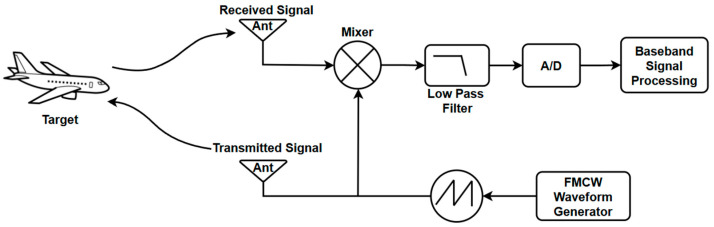
FMCW radar block diagram.

Figure 1 presents the block diagram of the FMCW radar system considered in this study. The system consists of a signal generator, transmitter, receiver, mixer, and an ADC. A frequency-modulated waveform is transmitted through the radar antenna and reflected from the target. The received signal is mixed with a copy of the transmitted signal to generate an IF beat signal, which contains both range- and velocity-related information. The digitized IF signal is subsequently processed in the digital domain to estimate the target parameters.

The front-end design and signal flow of the FMCW radar system were implemented in MATLAB Simulink (R2023b, MathWorks, Natick, MA, USA). Simulink is used to generate sequential bandwidth-encoded chirps using two FMCW Waveform blocks, which are then transmitted sequentially at each chirp duration using a Clock block. Phase noise is then applied to the generated signals, and gain and noise are added via the Transmitter block before being transmitted to the antenna stage. The HFSS-designed antenna parameters are entered into the TX Array block, and the directionality of the transmitted signal is determined. The signal, now transmitted through the air, passes through a Two-Ray channel and reaches the receiver antenna. The signal, which contains information about the distance, speed, and direction of the target object, reaches four sub-array antennas. The signal arriving at the antenna is divided into 4 different channels, each containing noise and gain. The signal from each subarray channel is vector-multiplied with a replica of the transmitted signal using a De-chirp block. Finally, the signals are passed through an IF low-pass filter, and the data is processed using MATLAB. The proposed hybrid chirp waveform and the associated signal processing chain used for range, velocity, and angle estimation are described in the following sections.

### 2.1. Hybrid Chirp Waveform Design

**Figure 2:**
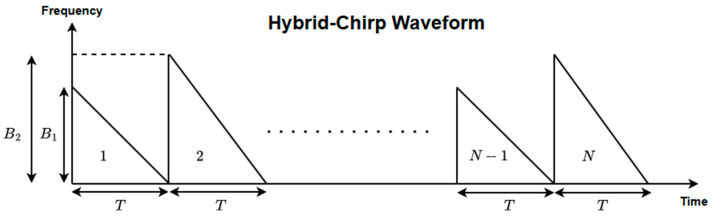
Proposed hybrid chirp waveform.

In this study, a hybrid sawtooth chirp waveform is employed to enable efficient velocity estimation within a short observation time. As illustrated in Figure 2, consecutive chirps have a constant duration T but different frequency sweep bandwidths, denoted by $B_{1}$ and $B_{2}$. Accordingly, successive chirps exhibit different sweep slopes (B/T) while maintaining identical time durations.

This waveform design introduces a controlled variation in the range-dependent component of the beat frequency without altering the Doppler component, which depends solely on the target velocity and carrier frequency. By exploiting this property, velocity estimation can be achieved using only two consecutive chirps, thereby avoiding the need for a large chirp sequence as required in conventional 2D-FFT-based approaches.

### 2.2. Signal Processing and Velocity Estimation

**Figure 3:**
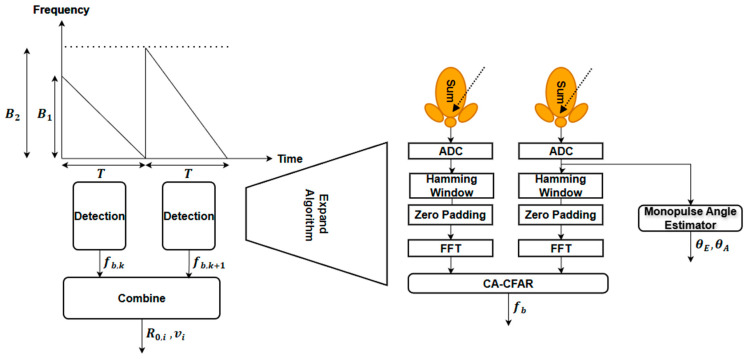
FMCW radar signal processing algorithm.

Following waveform transmission and reception, the IF signals corresponding to each chirp are processed according to the signal processing flow illustrated in Figure 3. To reduce spectral leakage around the fundamental beat frequency and suppress sidelobes, the time-domain IF signals are first windowed using a Hamming window. This operation improves frequency-domain resolution and enhances target detectability, particularly for mid-range targets [20].

Subsequently, a one-dimensional fast Fourier transform (1D-FFT) is applied to each windowed chirp signal to obtain the beat frequency spectrum. To eliminate spurious detections caused by noise and clutter, a constant false alarm rate (CFAR) detection algorithm is employed. In this work, cell-averaging CFAR (CA-CFAR) is selected due to its effectiveness in homogeneous noise environments [21]. After CFAR processing, the dominant beat frequencies corresponding to the target are identified for both chirps.

The beat frequency for the k-th chirp can be expressed as [3]

(1) $$f_{b,k}=f_{r}+f_{d}=\frac{2R_{0,k}}{c}\frac{B_{1}}{T}+\frac{2f_{c}v_{k}}{c},$$

while for the subsequent chirp it is given by

(2) $$f_{b,k+1}=\frac{2R_{0,k+1}}{c}\frac{B_{2}}{T}+\frac{2f_{c}v_{k}}{c},\;k=1,2,\ldots ,N,$$

(3) $$\frac{2R_{0,k}}{c}\frac{B_{1}}{T}{=f}_{b,k}-\frac{2f_{c}v_{k}}{c},$$

(4) $$\frac{2R_{0,k+1}}{c}\frac{B_{2}}{T}{=f}_{b,k+1}-\frac{2f_{c}v_{k}}{c},$$

(5) $$R_{0,k+1}=R_{0,k}+v_{k}T,\;\;\;\;\;R_{0,k}≅R_{0,k+1},$$

(6) $$\frac{B_{1}}{B_{2}}≅\frac{f_{b,k}-\frac{2f_{c}v_{k}}{c}}{f_{b,k+1}-\frac{2f_{c}v_{k}}{c}},$$

(7) $$v_{k}=\frac{c}{{2f}_{c}}\left(\frac{B_{2}f_{b,k}-B_{1}f_{b,k+1}}{B_{2}-B_{1}}\right).\;$$

In (1), $f_{r}$ is the range-induced beat frequency, $f_{d}$ is the Doppler frequency, $R_{0,k}$ denotes the target range, $v_{k}$ is the radial velocity, $f_{c}$ is the carrier frequency, and c is the speed of light. Since the Doppler frequency component $f_{d}$ depends only on the target velocity and carrier frequency, it remains identical for consecutive chirps. It is assumed that the target velocity does not change between two successive chirps or that any variation is smaller than the FFT velocity resolution. In contrast, the range-related frequency component $f_{r}$ is directly proportional to the chirp bandwidth, as shown in (6). As a result, changing the bandwidth between consecutive chirps introduces a measurable shift in the IF frequency associated with the target range by Equations (1) and (2). The chirp slope (S), IF bandwidth $\left(F_{IF_{max}}\right)$, sampling rate $\left(F_{S}\right)$, maximum unambiguous measurable velocities $\left(v_{max}\right)$, range resolution $\left(∆R\right)$ are of the FMCW radar is respectively given by [22]

(8) $$S=\frac{B}{T},$$

(9) $$F_{IF_{max}}=\frac{2SR_{max}}{c},$$

(10) $$F_{S}\geq 2F_{IF_{max}},$$

(11) $$v_{max}=\frac{\lambda_{c}}{4T},$$

(12) $$∆R=\frac{c}{2B},$$

and the range-dependent frequency component for the k-th chirp can be written as

(13) $$f_{r,k}=\frac{2R_{0,k}B_{\left(1+\left(k+1\right)mod2\right)}}{cT},\;k=1,2,\ldots ,N.$$

By exploiting the difference in beat frequencies resulting from the bandwidth variation, the range-dependent and Doppler components can be separated by using (6). Based on this separation, the target speed can be derived from (7) since the beat frequencies and bandwidth encoding are known.

For reliable velocity estimation, the IF frequency shift caused by the bandwidth difference must exceed both the FFT frequency resolution and the inverse chirp duration. This requirement can be expressed as

(14) $$\frac{2R_{0,k}\left|B_{1}-B_{2}\right|}{cT}>max\left(\frac{F_{s}}{N},\frac{1}{T}\right),$$

where $F_{s}$ denotes the sampling frequency and N is the FFT size. If this condition is not satisfied, the range-dependent frequency component cannot be clearly distinguished from the Doppler component, leading to degraded velocity estimation accuracy.

## 3. Monopulse Angle Estimation and Antenna Design

Monopulse radar systems provide accurate angle-of-arrival (AOA) estimation using a single snapshot, making them well suited for real-time tracking applications with strict computational and latency constraints. Unlike subspace-based DOA estimation techniques, monopulse systems are not directly dependent on the radar cross section (RCS) of the target and are therefore less sensitive to amplitude fluctuations in the received echo. As a result, monopulse methods can achieve high angular accuracy with minimal computational complexity [23,24].

The monopulse technique is implemented directly on the received FMCW signals within the same radar architecture, where sum and difference channels are formed for angle estimation. No separate pulse-based radar operation is used.

**Figure 4:**
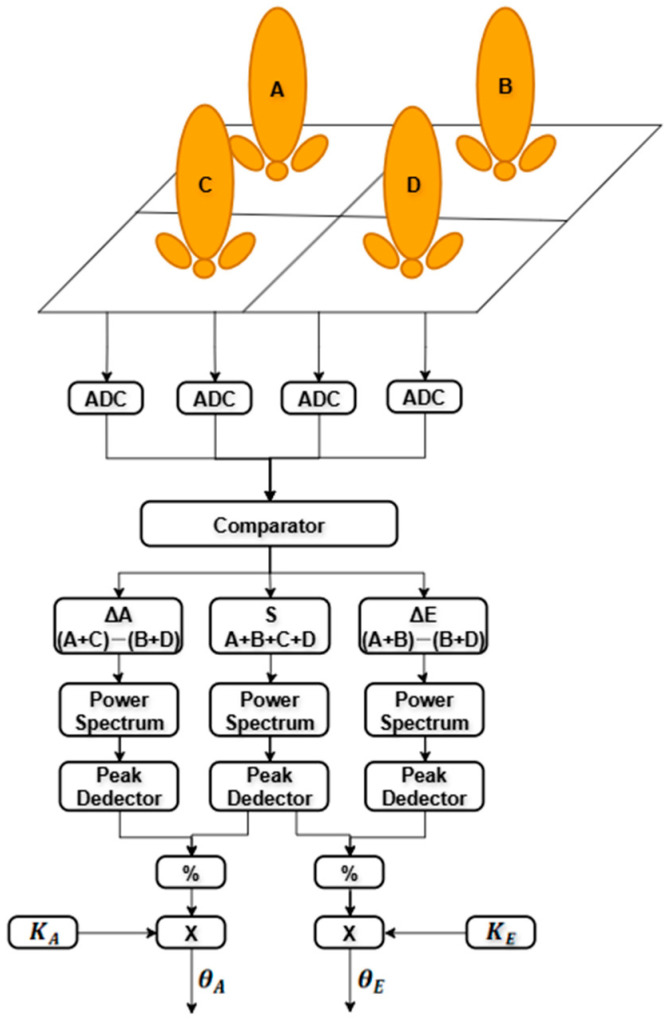
Amplitude comparison monopulse algorithm where A, B, C and D represent the subarrays of the antenna.

In amplitude-comparison monopulse systems, the antenna structure typically consists of symmetrically arranged sub-arrays. In this study, four sub-arrays are employed and positioned symmetrically around a reference axis, as illustrated in Figure 4. The angular position of the target is estimated by comparing the amplitudes of the signals received by each sub-array. 

**Figure 5:**
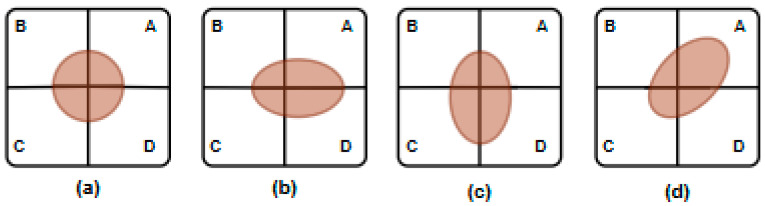
Two-dimensional amplitude projection according to target position: (**a**) target at the center axis of the antenna’s beam; (**b**–**d**) target is not at the center axis of the antenna’s beam.

When the target is located exactly on the boresight, the received signal amplitudes from all sub-arrays are equal, as shown in Figure 5a. If the target deviates from the boresight, the sub-array oriented toward the target direction receives a stronger signal, as illustrated in Figure 5b–d. These amplitude differences are exploited to generate an error signal related to the angular position of the target. The monopulse error signal can be defined as

(15) $$e=\frac{∆}{\Sigma }cos\varphi ,$$

where Δ and Σ denote the difference and sum signals, respectively, and φ represents the phase difference between the difference and sum channels. For small angular deviations around the boresight, this ratio exhibits an approximately linear relationship with the target angle, enabling precise angle estimation using simple linear processing.

In the proposed design, the sum and difference signals are generated digitally using the sub-array outputs sampled by the ADC. To reduce sidelobe levels and suppress unwanted interference, Taylor tapering is applied to the sum pattern, which enhances angular estimation performance by improving main-lobe dominance [25]. Thus, the voltage response of each sub-array is given by [26]

(16) $$V_{i}=\sum_{n}\sum_{m}w_{mn}e^{jk({md}_{x}sin\theta cos\phi +{nd}_{y}sin\theta sin\phi )}\;,\;\;\;\;\;i=A,B,C,D,$$

where $V_{i}$ represents the received voltage of each sub-array, $w_{mn}$ denotes the weighting coefficient of each antenna element, θ and ϕ spatial angle of a signal coming from a particular direction, $d_{x}$ and $d_{y}$ are the inter-element spacings in the x and y directions expressed in terms of wavelength, and k is the wavenumber. The antenna element indices are defined as:

- n=1,…,N and m=1,…,M for A
- n=N+1,…,2N and m=M+1,…,2M for B
- n=2N+1,…,3N and m=2M+1,…,3M for C
- n=3N+1,…,4N and m=3M+1,…,4M for D

with N=M= 6, corresponding to a 6 × 6 element configuration for each sub-array.

The sum and difference channel voltages are then formed as [23]

(17) $$V_{\Sigma }=V_{A}+V_{B}+V_{C}+V_{D},$$

(18) $$V_{\Delta_{E}}=\left(V_{A}+V_{B}\right)-(V_{C}+V_{D}),$$

(19) $$V_{\Delta_{A}}=\left(V_{A}+V_{C}\right)-\left(V_{B}+V_{D}\right).$$

where $\Delta_{A}$ and $\Delta_{E}$ represent the azimuth and elevation difference channels, respectively.

The monopulse ratio Δ/Σ alone does not directly yield the absolute angular position of the target. This ratio must be calibrated according to the specific antenna radiation pattern used in the system. This calibration introduces a proportionality constant, referred to as the calibration coefficient K, which maps the monopulse ratio to the corresponding angular deviation [24].

In this study, a symmetric 2 × 2 sub-array configuration is designed, where each sub-array consists of a 6 × 6 antenna element array. A shared-antenna topology is employed, allowing the same antenna elements to function for both transmission and reception. Detailed antenna parameters are provided in Table 1.

**Figure 6:**
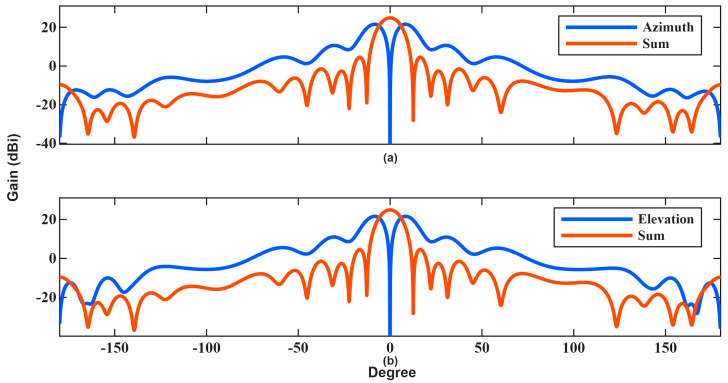
Designed antenna patterns: (**a**) azimuth difference and sum, (**b**) elevation difference and sum.

Electromagnetic modeling was performed via Ansys HFSS (neglecting radome/platform interactions), and the resulting far-field radiation patterns were imported into an FMCW radar simulator to evaluate performance. The azimuth and elevation difference and summation patterns of the designed antenna are shown in Figure 6.

**Figure 7:**
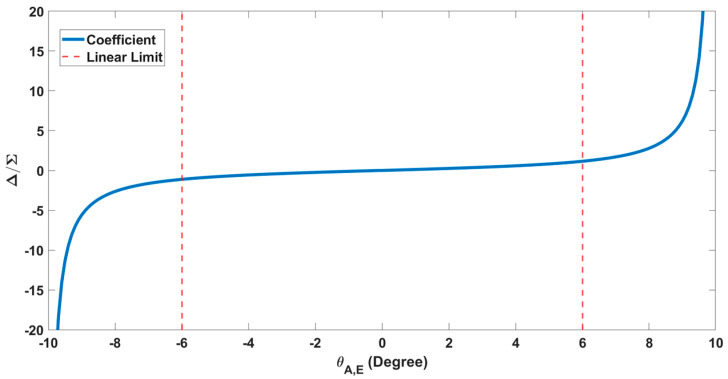
Calibration coefficients (K) extracted at 0.1° intervals.

The monopulse ratios $\Delta_{A}/\Sigma$ and $\Delta_{E}/\Sigma$ are extracted from the simulated antenna patterns at angular intervals of 0.1°. These discrete values are subsequently interpolated to achieve more precise angle estimation. The resulting calibration coefficients for all angular directions are shown in Figure 7.

The quantitative monopulse sensitivity is calculated as 0.1002 V/V/deg within linear region from Figure 7.

**Figure 8:**
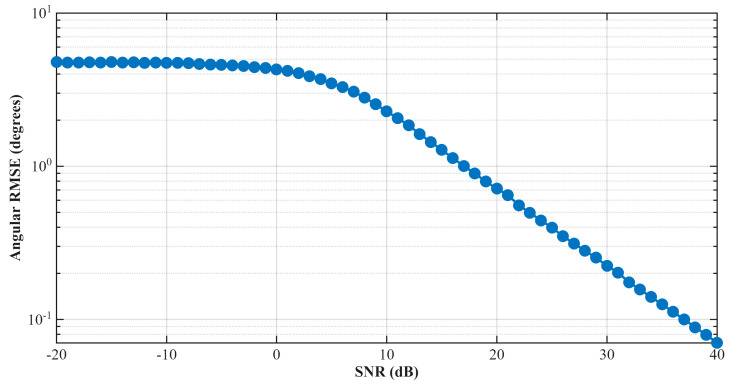
Angular RMSE versus SNR of the monopulse system when the target at antennas boresight (0°).

Figure 8 illustrates the angular RMSE of the monopulse tracking system versus SNR. The Monte Carlo simulations utilize the extracted effective monopulse sensitivity of 0.1002 V/V/deg within the $\pm 6^{{}^{\circ}}$ operational field of view. The scenario runs with 10000 Monte Carlo simulations in the case where the target is within the antenna’s boresight. At low SNR levels, the tracking error deliberately saturates due to the defined physical tracking limits, whereas it smoothly converges to the theoretical accuracy bounds (e.g., $\approx 0.0725^{{}^{\circ}}$ SNR at 40 dB) as the signal quality improves. The approximate RMSE equation, which theoretically depends on the SNR and monopulse slope, is given in (20) where $k_{m}$ refers to quantitative monopulse sensitivity.

(20) $$RMSE\approx \frac{1}{k_{m}\sqrt{2SNR_{linear}}}$$

The azimuth ($\theta_{A}$) and elevation ($\theta_{E}$) angles of the target are then calculated as

(21) $$\theta_{A}=K_{A}\left(\frac{{P_{\Delta }}_{A}}{P_{\Sigma }}\right)\cos\left(\Delta \varphi \left(V_{{∆}_{A}},V_{\Sigma }\right)\right)$$

(22) $$\theta_{E}=K_{E}\left(\frac{{P_{\Delta }}_{E}}{P_{\Sigma }}\right)\cos\left(\Delta \varphi \left(V_{{∆}_{E}},V_{\Sigma }\right)\right)$$

where P denotes the power of the corresponding channel signal. The calibration coefficients are defined as

(23) $$K_{A}=\frac{\theta_{A}}{{∆}_{A}/\Sigma }$$

(24) $$K_{E}=\frac{\theta_{E}}{{∆}_{E}/\Sigma }$$

Since the antenna array is designed symmetrically in both azimuth and elevation planes, the calibration coefficients satisfy

(25) $$K_{A}\approx K_{E}$$

The inter-element spacing is selected as $d_{x}=d_{y}=\lambda_{c}/2$, while the distance between adjacent sub-arrays is set to 3$\lambda_{c}$. As the spacing between sub-arrays increases, the angular region over which the monopulse ratio remains linear decreases [24]. For the proposed antenna design, the linear operating region is approximately [−6°, 6°], as observed in Figure 7. Because the antenna beam is not electronically steered toward the target, accurate angle estimation needs to be performed within this linear region.

## 4. Kalman-Based Target Tracking

Radar measurements are inherently affected by sensor noise, environmental disturbances, and target maneuvering effects. Therefore, tracking filters are required to obtain optimal state estimates from noisy measurements. The Kalman filter provides an optimal solution for linear systems with Gaussian noise assumptions. However, real-world radar tracking problems are typically nonlinear due to the nonlinear relationship between the measured quantities and the target state [27]. To address this issue, linearization-based Kalman filtering techniques are commonly employed.

In this study, the tracking performance of two widely used nonlinear Kalman filtering approaches is investigated: the EKF and UKF. Both methods are applied to track the target’s spatial position and motion using range, velocity, azimuth, and elevation measurements provided by the FMCW radar system.

**Figure 9:**
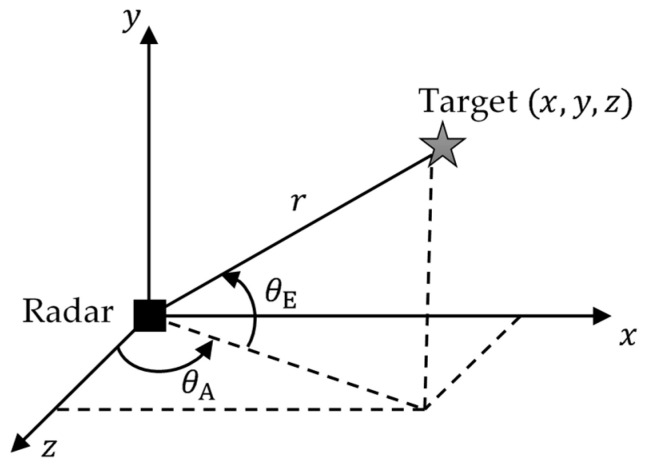
Spatial location of the target.

Figure 9 illustrates the spatial configuration of the target with respect to the radar sensor. The radar measurements are obtained in spherical coordinates and transformed into Cartesian space through nonlinear measurement equations, which constitute the observation model of the tracking system.

### 4.1. Motion and Measurement Models

The radar sensor provides measurement data in spherical coordinates, which are related to the target state in Cartesian coordinates through nonlinear transformation equations. The measurement vector is defined as [28]

(26) $$h=\left[\begin{array}{c} r_{n}\\ {\dot{r}}_{n}\\ \theta_{E_{n}}\\ \theta_{A_{n}} \end{array}\right]=\left[\begin{array}{c} \sqrt{x_{n}^{2}+y_{n}^{2}+z_{n}^{2}}\\ \frac{{\dot{x}}_{n}x_{n}+{\dot{y}}_{n}y_{n}+{\dot{z}}_{n}z_{n}}{\sqrt{x_{n}^{2}+y_{n}^{2}+z_{n}^{2}}}\\ \tan^{-1}(\frac{y_{n}}{x_{n}})\\ \sin^{-1}\left(\frac{z_{n}}{\sqrt{x_{n}^{2}+y_{n}^{2}+z_{n}^{2}}}\right) \end{array}\right],$$

where $r_{n}$ and ${\dot{r}}_{n}$ denote the target range and radial velocity, respectively. The variables $x_{n}$, $y_{n}$, $z_{n}$ represent the target position in Cartesian coordinates, while ${\dot{x}}_{n}$, ${\dot{y}}_{n}$, ${\dot{z}}_{n}$ denote the corresponding velocity components. The index n represents the measurement sequence.

The target motion is modeled using a Newtonian constant-acceleration model. Although real targets do not strictly follow constant acceleration, deviations from the nominal motion model are captured through process noise. The discrete-time state transition model is expressed as [29]

(27) $$x_{k+1}=Fx_{k}+w_{k}.$$

where $x_{k}$ is the state vector at time step k, F is the state transition matrix, and $w_{k}\sim N$(0,Q) represents zero-mean Gaussian process noise.

The state transition matrix corresponding to the constant-acceleration model is defined as

(28) $$F=I_{3}⨂\left[\begin{array}{ccc} 1 & dt & \frac{dt^{2}}{2}\\ 0 & 1 & dt\\ 0 & 0 & 1 \end{array}\;\right],$$

where $I_{3}$ is the 3 × 3 identity matrix, ⨂ denotes the Kronecker product, and dt is the time interval between successive measurements.

The process noise covariance matrix is given by

(29) $$Q=I_{3}⨂\left[\begin{array}{ccc} \frac{dt^{4}}{4} & \frac{dt^{3}}{2} & \frac{dt^{2}}{2}\\ \frac{dt^{3}}{2} & dt^{2} & dt\\ \frac{dt^{2}}{2} & dt & 1 \end{array}\right]\sigma_{a}^{2},$$

where $\sigma_{a}^{2}$ represents the variance of the process noise. Generally, $\sigma_{a}$ chosen interval $0.5∆a_{M}\leq \sigma_{a}\leq ∆a_{M}$. $∆a_{M}$ refers to magnitude of the maximum acceleration increment along the sampling interval T.

The measurement noise covariance matrix is defined as

(30) $$R_{n}=diag\left(\sigma_{r}^{2},\sigma_{\dot{r}}^{2},\sigma_{\theta_{Az}}^{2},\sigma_{\theta_{El}}^{2}\right),$$

assuming that range, velocity, azimuth, and elevation measurement errors are statistically independent.

The initial state covariance matrix is defined as

(31) $$P_{k}=\left[\begin{array}{ccc} p_{k} & p_{k\dot{k}} & p_{k\ddot{k}}\\ p_{\dot{k}k} & p_{\dot{k}} & p_{\dot{k}\ddot{k}}\\ p_{\ddot{k}k} & p_{\ddot{k}\dot{k}} & p_{\ddot{k}} \end{array}\right],\;\;\;\;k=x,y,z,$$

and the overall initial covariance matrix is constructed as a block-diagonal matrix

(32) $$P_{0,0}=blkdiag\left(P_{x},P_{y},P_{z}\right),$$

where $p_{k}$, $p_{\dot{k}}$, and $p_{\ddot{k}}$ are the variance of the position, velocity and acceleration of each Cartesian coordinate (x,y,z) respectively, and the off-diagonal terms denote the corresponding covariances [30].

### 4.2. Extended Kalman Filter (EKF)

The EKF addresses nonlinear estimation problems by analytically linearizing the nonlinear system equations around the current state estimate. This linearization is performed using first-order Taylor series expansion and requires the computation of Jacobian matrices [28].

In this study, the state transition model is linear. Therefore, the Jacobian of the state transition function is identical to the state transition matrix F. However, the observation model defined in (26) is nonlinear, and its Jacobian matrix needs to be computed. The general Jacobian expressions are given by [30]

(33) $$\frac{\partial f}{\partial x}=\left[\begin{array}{ccc} \frac{\partial f_{1}}{\partial x_{1}} & \ldots & \frac{\partial f_{1}}{\partial x_{n}}\\ \vdots & \ddots & \vdots \\ \frac{\partial f_{m}}{\partial x_{1}} & \ldots & \frac{\partial f_{m}}{\partial x_{n}} \end{array}\right],$$

(34) $$\frac{\partial h}{\partial x}=\left[\begin{array}{ccc} \frac{\partial h_{1}}{\partial x_{1}} & \ldots & \frac{\partial h_{1}}{\partial x_{n}}\\ \vdots & \ddots & \vdots \\ \frac{\partial h_{m}}{\partial x_{1}} & \ldots & \frac{\partial h_{m}}{\partial x_{n}} \end{array}\right],$$

where f denotes the state transition function that describes the target motion model, while h represents the nonlinear measurement function that maps the Cartesian state vector to radar measurements in spherical coordinates, as defined in (26).

### 4.3. Unscented Kalman Filter (UKF)

The Unscented Kalman Filter (UKF) provides an alternative approach to nonlinear estimation without requiring explicit analytical linearization. Instead, UKF employs a statistical linearization technique known as the Unscented Transform (UT). In the UKF framework, a deterministic set of sigma points is generated around the current state estimate based on the state covariance. These sigma points are propagated through the nonlinear state transition and measurement functions, resulting in transformed sigma points that approximate the posterior state distribution [19]. The number of sigma points is determined by the input distribution. Since both negative and positive points will be selected around the mean, 2L+1 points should be selected. Since this design includes nine system states, 19 sigma points were selected.

(35) $$\lambda =\alpha^{2}\left(L+\kappa \right)-L$$

where L is the dimension of the system state vector, α determines the spread of the sigma points around the mean. A higher α provides a larger spread of the sigma points around the mean and reduces the effect of high order terms. Therefore, α is set to 0.01. κ is a secondary scaling parameter used to ensure that the variance remains positively defined that is generally set to 0 [27].

(36) $$X_{n,n\;}^{\left(0\right)}={\hat{x}}_{n,n},$$

(37) $$X_{n,n=}^{\left(i\right)}{\hat{x}}_{n,n}+{\left(\sqrt{\left(L+\lambda \right)P_{n,n}}\right)}_{i},\;i=1,\ldots ,L,$$

(38) $$X_{n,n=}^{\left(i\right)}{\hat{x}}_{n,n}-{\left(\sqrt{\left(L+\lambda \right)P_{n,n}}\right)}_{i-N},\;i=L+1,\ldots ,2L,$$

After the sigma points calculated, sigma points weights should be calculated:

(39) $$w_{0}^{\left(m\right)}=\frac{\lambda }{L+\kappa },$$

(40) $$w_{0}^{\left(c\right)}=\frac{\lambda }{L+\lambda }+(1-\alpha^{2})+\beta ,$$

(41) $$w_{i}=\frac{1}{2\left(L+\lambda \right)},\;i=1,.\;.\;.,2L,\;i>0,$$

where $w_{0}^{\left(m\right)}$ is the weight for the first sigma point $X_{n,n\;}^{\left(0\right)}$ when computing the weighted mean, $w_{0}^{\left(c\right)}$ is the weight for the first sigma point $X_{n,n\;}^{\left(0\right)}$ when computing the weighted covariance, and $w_{i}$ is the weight for the other sigma points ${X_{n,n}}^{\left(i\right)}$, with i>0 when computing the weighted mean or covariance. In addition, β is used to incorporate prior knowledge of the distribution of the input random variable. For Gaussian distributions, β= 2 is optimal [27].

Using weighted combinations of these transformed sigma points, the mean and covariance of the state and measurement distributions are computed. While EKF achieves only first-order accuracy due to linearization, UKF captures the mean and covariance up to second-order accuracy for Gaussian inputs. Although UKF incurs higher computational complexity, it provides improved robustness and convergence properties in strongly nonlinear systems [17].

## 5. Results

This section presents the simulation results obtained to evaluate the performance of the proposed FMCW radar processing framework. The analysis focuses on three main aspects: (a) the effectiveness of the proposed hybrid chirp waveform for velocity estimation, (b) the performance of monopulse-based angle estimation under realistic antenna and SNR conditions, and (c) a comparative evaluation of EKF and UKF for target tracking.

### 5.1. Simulation Setup

**Figure 10:**
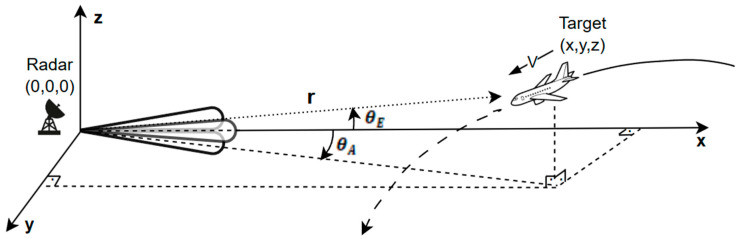
Target motion and radar appearance consistent with the constant acceleration model.

The FMCW radar, target, and environmental models were implemented in MATLAB Simulink. The radar sensor is assumed to be stationary and located at the origin (0, 0, 0), while the target at (x, y, z) follows a constant-acceleration motion model, as illustrated in Figure 10.

The FMCW radar front-end parameters were selected to represent a realistic short- to medium-range sensing configuration while enabling reliable detection and tracking of high-velocity targets. The operating frequency was set to 11 GHz, providing a suitable compromise between antenna aperture size, propagation characteristics, and Doppler sensitivity. A chirp duration of 2 ms was chosen to ensure a sufficiently high update rate for fast-moving targets while maintaining manageable processing latency. The transmitted power was set to 50 dBm to guarantee adequate SNR within the considered range, whereas the noise floor and receiver noise figure were selected to model practical hardware limitations rather than ideal conditions.

The antenna geometry was designed to support monopulse angle estimation. The antenna element spacing within each subarray was set to half the wavelength to avoid grating lobes, while the spacing between subarrays was chosen as three wavelengths to achieve sufficient angular sensitivity. The resulting field of view was limited to ±6°, which corresponds to the linear operating region of the antenna pattern used for accurate monopulse processing. The simulation parameters of the FMCW radar front-end are summarized in Table 2.

In addition to the hardware and waveform parameters, the signal processing chain includes beat signal generation, spectral analysis, CFAR-based detection, monopulse-based angle extraction, and measurement extraction from the simulated IF signals. Noise is modeled as additive white Gaussian noise consistent with the specified noise floor and receiver noise figure. Both hybrid and conventional chirp cases are evaluated under identical conditions to ensure fair comparison. For tracking analysis, EKF and UKF are implemented using the same motion and noise assumptions, and their performance is evaluated based on estimation accuracy, convergence behavior, and RMSE metrics over multiple Monte Carlo runs. Further implementation details and performance evaluations are provided in the following subsections.

### 5.2. Velocity Estimation Performance

To clearly demonstrate the limitations of conventional FMCW velocity estimation and to highlight the advantages of the proposed hybrid chirp approach, two representative scenarios are considered. In the first scenario, consecutive chirps with identical sweep bandwidths are transmitted to illustrate the inherent ambiguity in velocity estimation. In the second scenario, the proposed hybrid chirp waveform with different bandwidths is employed to show how this ambiguity can be resolved using only two chirps.

#### 5.2.1. Scenario 1: Equal Bandwidth Chirps

**Figure 11:**
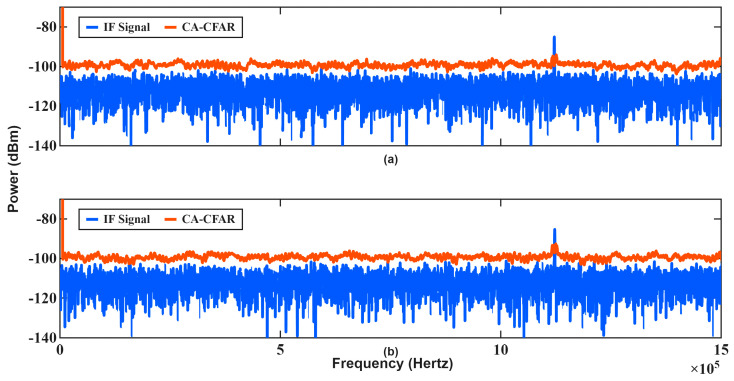
IF spectrums and threshold for the first measurement in Table 3: (**a**) first chirp, (**b**) second chirp.

In the first scenario, the radar transmits consecutive chirps with identical bandwidths of 300 MHz ($B_{1}=B_{2}$). The corresponding estimation results are summarized in Table 3, and the IF spectra of the first measurement are illustrated in Figure 11.

As shown in Figure 11, the IF spectra of consecutive chirps exhibit nearly identical frequency components. Since the sweep slopes are equal, both the range-dependent and Doppler-dependent terms collapse into a single frequency contribution, as predicted by (1) and (2). Consequently, the Doppler frequency cannot be isolated from the range component, resulting in ambiguity in velocity estimation. This behavior is clearly reflected in Table 3, where angle estimation remains accurate while velocity estimation fails for all test cases. The monopulse angle estimation relies primarily on amplitude ratios and is therefore insensitive to this ambiguity, whereas velocity extraction critically depends on separating Doppler-induced shifts from range-induced frequency components.

In Figure 11, slight differences can be in the maximum IF frequencies of successive chirps; these variations stem from noise and phase jitter effects rather than deterministic waveform properties. Because these effects are random, they do not provide reliable velocity information. As a result, conventional FMCW processing using identical chirps becomes ineffective for high-speed targets, particularly at short ranges where Doppler coupling strongly biases the beat frequency. This scenario demonstrates the fundamental limitation of classical FMCW velocity estimation when no waveform diversity is introduced between consecutive chirps.

#### 5.2.2. Scenario 2: Hybrid Chirp Waveform

**Figure 12:**
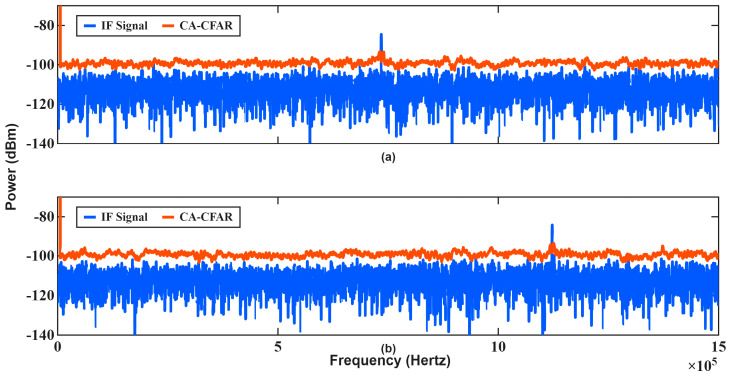
IF spectrums and threshold for the first measurement in Table 4: (**a**) first chirp (200 MHz), (**b**) second chirp (300 MHz).

In the second scenario, consecutive chirps with bandwidths of 200 MHz ($B_{1}$) and 300 MHz ($B_{2}$) are transmitted. The estimation results are summarized in Table 4, while the corresponding IF spectra are shown in Figure 12.

Unlike Scenario 1, a clear separation between the range-dependent and Doppler-dependent frequency components is observed due to the bandwidth difference between consecutive chirps, as predicted by (1) and (2). This frequency shift enables independent estimation of target range and velocity using only two chirps. The range shift between successive chirps is shown in (5). Using Equation (1), the effect of the target’s consecutive chirps distance shift on the IF frequency shift and the Doppler shift caused by the target’s velocity were calculated separately. The frequency shift due to the range shift is 4 kHz, while the Doppler frequency shift is 150 kHz. Since the frequency shift caused by the range shift is negligible compared to the Doppler frequency shift, it can be assumed that the distance between successive chirps in Equation (5) is approximately the same.

The results in Table 4 also demonstrate that accurate velocity measurements are obtained over a wide range of target distances and velocities, including very high-speed targets. Notably, reliable measurements are achieved for velocities around Mach 8, confirming that the proposed method effectively improves the maximum unambiguous velocity limitation inherent in conventional 2D-FFT-based approaches. The unsuccessful measurements occur in the 11th case, where the target lies outside the antenna field of view and propagates through low-gain regions of the antenna pattern, and in case 13, due to the maximum measurable velocity limitation resulting from the IF frequency visibility limitations. This failure in case 11 was attributed to antenna coverage limitations rather than to the proposed waveform or signal processing method.

It should be noted that the CFAR detection threshold was configured with a false alarm probability ($p_{fa}$) of 10^−7^, which restricts detections to targets with sufficiently high SNR. In the proposed velocity estimation method, since high velocities are no longer dependent on short chirp durations, longer chirps can be discarded, allowing for more listening and reducing the necessary SNR concern. In [31,32], the integration SNR gain in this long-term data collection is indicated. However, range and velocity measurements may still not be possible for targets passing through low-gain regions of the antenna pattern.

Thanks to this integration or FFT gain, angular information is obtained by using the amplitude of the dominant spectral peak in the sum and difference channels. Even if reliable range and velocity estimates cannot be obtained because of the CA-CFAR threshold, the approximate spatial orientation of the target relative to the radar remains observable, enabling angular tracking under relatively low SNR conditions. This behavior is an advantage for early target positioning and tracking.

**Figure 13:**
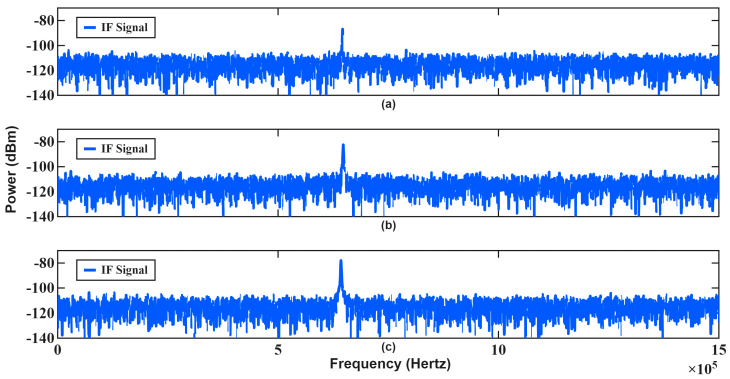
IF spectrums of channels for the third measurement in Table 4: (**a**) elevation channel, (**b**) azimuth channel, (**c**) summation channel.

Figure 13 illustrates the azimuth difference, elevation difference, and summation channel spectra for the third measurement in Table 4. The elevation peak is approximately −89.5 dBm, the azimuth peak is −82 dBm, and the sum channel peak is approximately −78 dBm. Angle information is obtained using the differences between these peak amplitudes, Equations (21) and (22), and the calibration coefficient, Equations (23) and (24), as shown in the graph in Figure 7. In general, Equations (17)–(19) are used to calculate the amplitude differences between the sum, azimuth and elevation channels.

These results highlight an important feature of the proposed frame system: distance and velocity information are obtained with two chirps, while angle information is obtained with a single chirp. This feature allows the entire system structure to obtain the necessary information in short periods of time.

### 5.3. Kalman Filter Tracking Performance

While the proposed FMCW waveform and monopulse processing enable accurate instantaneous range, velocity, and angle estimation, real-world radar measurements remain affected by noise, model uncertainty, and target maneuvering. For high-speed targets in particular, small measurement errors may rapidly accumulate over time, leading to degraded tracking performance if no temporal filtering is applied.

To address this issue, the estimated range, velocity, azimuth, and elevation measurements are subsequently processed by Kalman-based tracking algorithms. In this study, both the EKF and UKF are employed to assess their effectiveness in fusing noisy measurements and maintaining robust target state estimates over time. The target motion is modeled using a constant-acceleration assumption, and the initial state parameters are listed in Table 5.

In addition to the deterministic initial state values given in Table 5, the initial state uncertainty and measurement noise characteristics were explicitly defined to ensure a consistent and reproducible tracking setup. The initial covariance matrix was selected as

(42) $$P_{0,0}=I_{3}⨂\left[\begin{array}{ccc} 225 & 0 & 0\\ 0 & 10000 & 0\\ 0 & 0 & 0.008 \end{array}\right]$$

which represents substantial initial uncertainty in position, velocity, and acceleration along all Cartesian axes. The standard deviation of the range measurement error was set to $\sigma_{r}=$ 3 m, while the velocity measurement error standard deviation was chosen as $\sigma_{v}=$ 50 m/s, and the process noise standard deviation was set to $\sigma_{a}=2\;m/s^{3}$. The standard deviations of the azimuth and elevation angle measurement errors were both selected as 0.2° (0.0035 rad), which is consistent with practical monopulse-based angle estimation accuracy.

To comprehensively evaluate the sensitivity and robustness of the tracking filters, two representative tracking scenarios are considered. The first scenario reflects favorable conditions in which the initial filter states are close to the true target state, while the second scenario intentionally introduces large initial estimation errors to examine filter convergence behavior under challenging conditions. The convergence times and errors of the filters were determined for both scenarios by running 1000 Monte Carlo simulations.

#### 5.3.1. Scenario 1: Initial Conditions Close to True State

In the first tracking scenario, the initial filter states are selected close to the true target state, as summarized in Table 6. This scenario represents an idealized yet practically relevant situation in which coarse prior information about the target is available.

**Figure 14:**
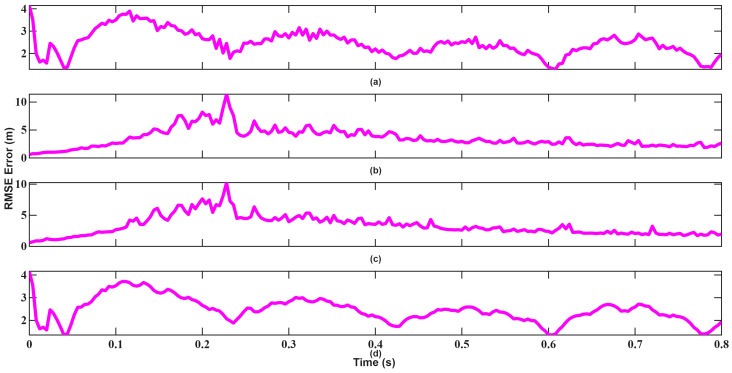
Position RMSEs of EKF for scenario 1: (**a**) x-axis, (**b**) y-axis, (**c**) z-axis, (**d**) Total error.

**Figure 15:**
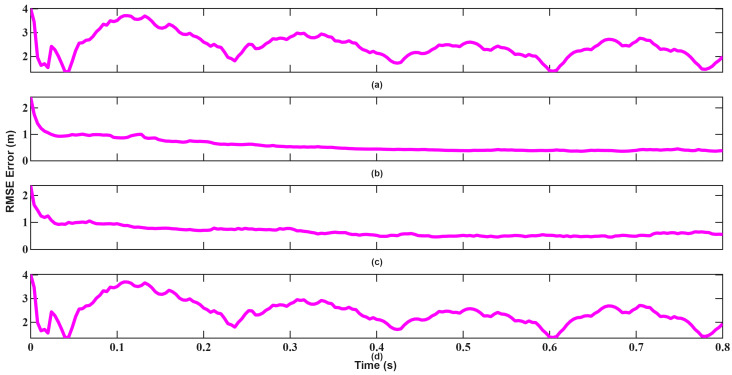
Position RMSEs of UKF for scenario 1: (**a**) x-axis, (**b**) y-axis, (**c**) z-axis, (**d**) Total error.

**Figure 16:**
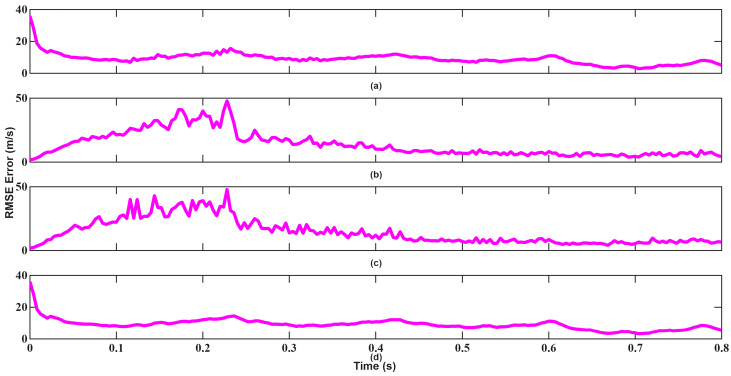
Velocity RMSEs of EKF for scenario 1: (**a**) x-axis, (**b**) y-axis, (**c**) z-axis, (**d**) Total error.

**Figure 17:**
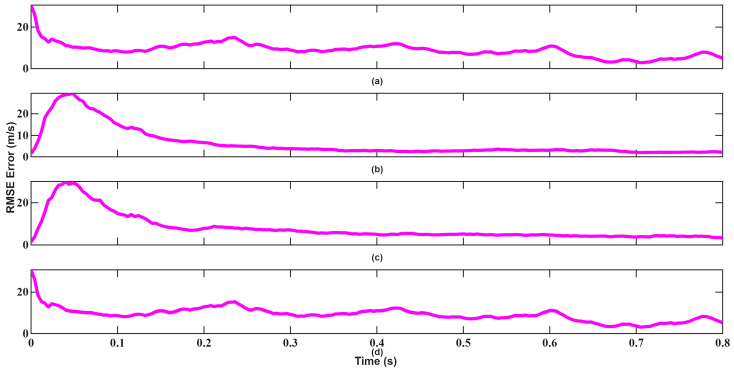
Velocity RMSEs of UKF for scenario 1: (**a**) x-axis, (**b**) y-axis, (**c**) z-axis, (**d**) Total error.

**Figure 18:**
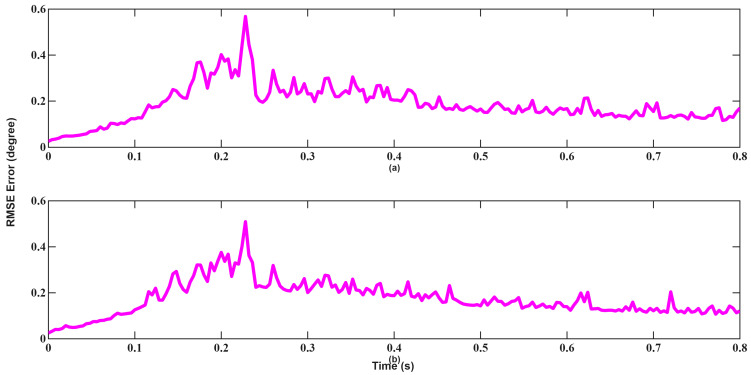
Angle RMSEs of EKF for scenario 1: (**a**) Azimuth, (**b**) Elevation.

**Figure 19:**
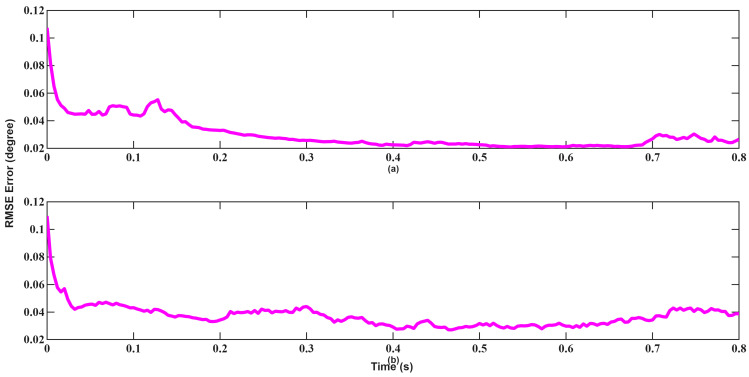
Angle RMSEs of UKF for scenario 1: (**a**) Azimuth, (**b**) Elevation.

The resulting position, velocity, and angle tracking estimation errors are shown in Figure 14, Figure 15, Figure 16, Figure 17, Figure 18 and Figure 19. As illustrated in Figure 14 and Figure 15, although UKF converges faster and with small errors, EKF also attempts to converge rapidly in position tracking when initialized near the true state. The estimation errors in the x, y, and z axes remain small for both filters, indicating that under mild nonlinear conditions, EKF also remains effective. However, UKF consistently achieves slightly lower steady-state errors, particularly in the y and z directions. This improvement stems from UKF’s ability to propagate nonlinear transformations more accurately using sigma points rather than linear approximations.

Velocity tracking performances shown in Figure 16 and Figure 17 further highlights this behavior. Since the velocity measurement equation is strongly nonlinear, EKF’s first-order linearization introduces bias, resulting in larger oscillations. UKF maintains smoother convergence and lower error variance by capturing second-order statistics. As seen, although UKF made more mistakes in the beginning, recovered much faster and converged.

In the angle tracking results shown in Figure 18 and Figure 19, UKF performs significantly better than EKF. Especially at medium distances, a slight deviation in angle will make target tracking quite difficult. UKF exhibits faster stabilization and lower jitter, particularly during transition periods. Furthermore, EKF still failed to converge at 0.8 s. Overall, the results from Scenario 1 confirm that while EKF demonstrates adequate performance under suitable initial conditions, UKF consistently provides better numerical stability and accuracy.

#### 5.3.2. Scenario 2: Poor Initial Conditions

In the second tracking scenario, the initial filter states are deliberately selected far from the true target state, as detailed in Table 7. This case emulates realistic operational conditions in which little or no prior information about the target is available.

**Figure 20:**
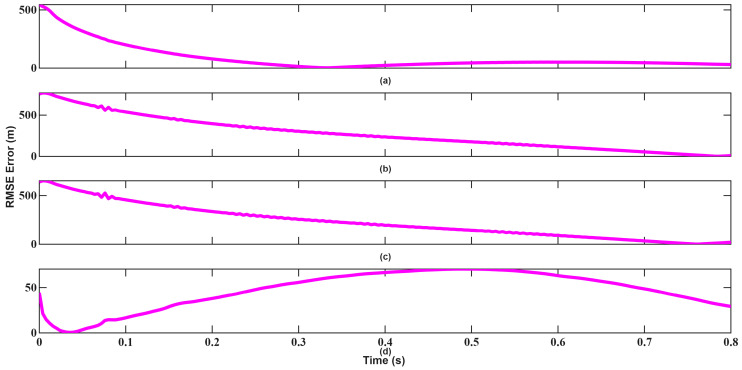
Position RMSEs of EKF for scenario 2: (**a**) x-axis, (**b**) y-axis, (**c**) z-axis, (**d**) total error.

**Figure 21:**
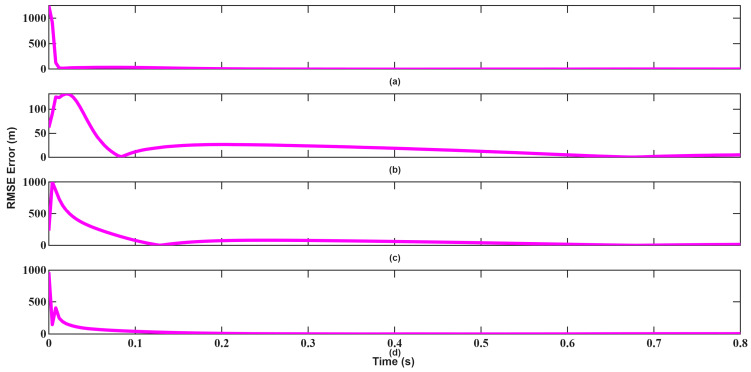
Position RMSEs of UKF for scenario 2: (**a**) x-axis, (**b**) y-axis, (**c**) z-axis, (**d**) total error.

**Figure 22:**
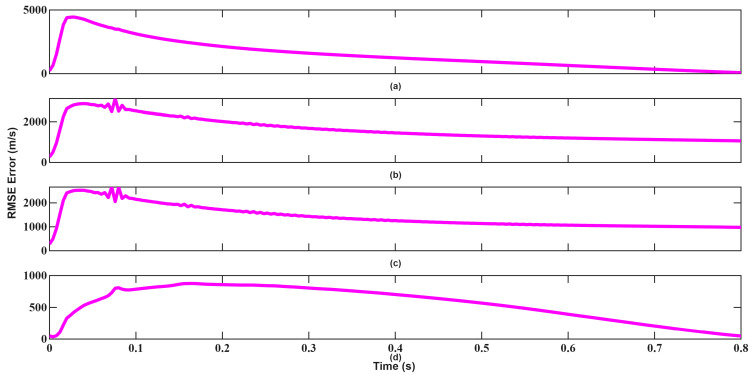
Velocity RMSEs of EKF for scenario 2: (**a**) x-axis, (**b**) y-axis, (**c**) z-axis, (**d**) total error.

**Figure 23:**
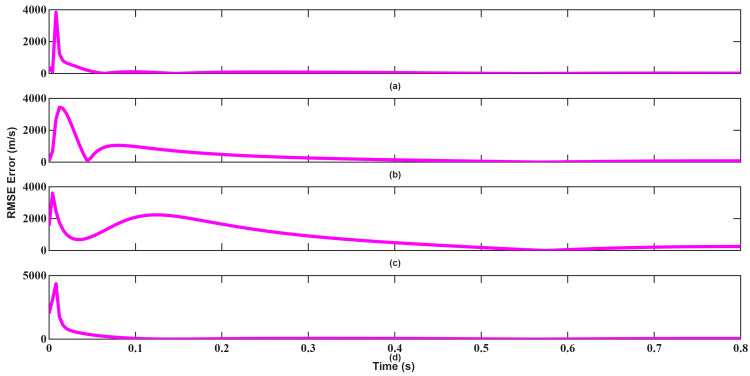
Velocity RMSEs of UKF for scenario 2: (**a**) x-axis, (**b**) y-axis, (**c**) z-axis, (**d**) total error.

**Figure 24:**
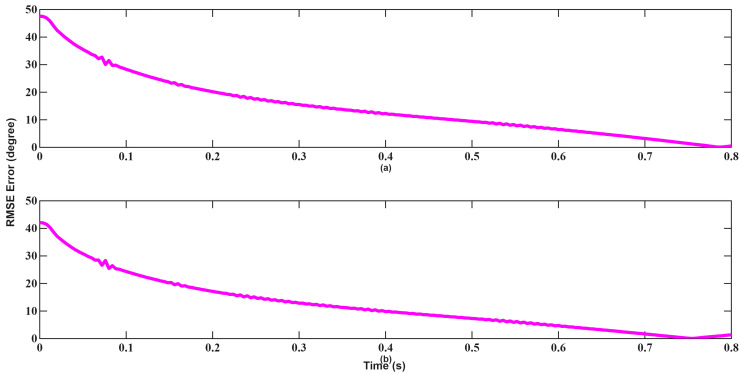
Angle RMSEs of EKF for scenario 2: (**a**) Azimuth, (**b**) Elevation.

**Figure 25:**
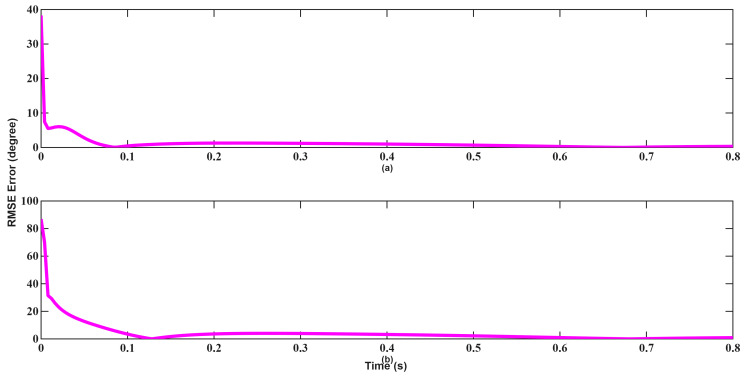
Angle RMSEs of UKF for scenario 2: (**a**) Azimuth, (**b**) Elevation.

The resulting tracking errors are presented in Figure 20, Figure 21, Figure 22, Figure 23, Figure 24 and Figure 25. As shown in Figure 20 and Figure 21, EKF requires more than 0.3 s to converge in position, which constitutes a significant limitation for high-velocity target tracking. Conversely, UKF converges to the true position within approximately 0.1 s, demonstrating faster recovery from large initialization errors.

Velocity tracking results shown in Figure 22 and Figure 23 further highlight this contrast. EKF fails to exhibit iterative convergence behavior caused by accumulated linearization errors, whereas UKF stabilizes rapidly after a short transient period and maintains low estimation error throughout the tracking interval.

A similar trend is observed in angle tracking shown in Figure 24 and Figure 25. Although EKF initially yields lower error, its convergence remains slow and inconsistent. UKF achieves near-zero angular error within approximately 0.1 s, highlighting its higher robustness and convergence characteristics in the presence of poor initial estimates.

The results achieved from Scenario 2 highlight the importance of nonlinear filtering in high-speed radar tracking. While EKF remains computationally efficient, UKF offers substantially improved convergence speed, stability, and robustness in realistic scenarios involving high velocity and nonlinear measurement models.

## 6. Discussion

This study addresses the challenges of high-velocity target detection and tracking using FMCW radar systems operating at short and medium ranges. Comparative summary of representative FMCW radar-based velocity estimation methods reported in the literature is provided in Table 8. Conventional FMCW velocity estimation techniques, primarily based on sawtooth or triangular waveforms combined with the 2D-FFT method, rely on a large number of chirps to improve velocity resolution, which inherently increases data acquisition time and introduces a strict maximum unambiguous velocity limitation. As demonstrated in previous studies [33,34], this limitation confines such approaches to low-speed targets, particularly in automotive radar applications. Even when short chirp durations and high carrier frequencies are employed to improve resolution and maximum velocity, as reported in [35], even when using multiple and short-duration chirps and high carrier frequencies to improve resolution and maximum speed, the maximum unambiguous velocity still remains limited to relatively low values.

Several attempts have been made to overcome this limitation. In [36], high-speed targets were detected by significantly shortening the chirp duration; however, this solution increases the required IF bandwidth, system noise, and ADC sampling rate, making it impractical for many real-world systems. Similarly, [3] employed a three-segment triangular waveform to achieve low velocity estimation error using only three chirps, but the maximum velocity limitation was not fully resolved. Moreover, Doppler shifts induced by high-speed, close-range targets introduced additional ambiguities in range and velocity estimation, necessitating further processing stages.

In contrast, the proposed hybrid chirp waveform with bandwidth coding fundamentally alters the relationship between range-dependent and Doppler-dependent frequency components. By maintaining a constant chirp duration while varying the sweep bandwidth between consecutive chirps, the proposed method enables reliable separation of range and velocity information using only two chirps. Simulation results confirm that this approach improves the maximum unambiguous velocity constraint. As summarized in Table 8, the proposed method achieves competitive velocity accuracy while significantly reducing data collection time compared to existing approaches.

Beyond velocity estimation, this work also demonstrates the effectiveness of amplitude-comparison monopulse angle estimation for fast single-target scenarios. Unlike subspace-based DOA estimation methods, which require multiple snapshots and incur high computational cost, the monopulse approach provides angular information using a single snapshot with minimal processing overhead. Simulation results show that accurate angle estimation is achievable when the target lies within the linear region of the antenna pattern and sufficient SNR is available. At low SNR levels, angular accuracy degrades as expected; however, approximate angular information can still be extracted, enabling continued target tracking even when range and velocity measurements become unreliable.

The integration of EKF and UKF into the proposed framework further highlights the importance of nonlinear filtering in high-speed radar tracking. As demonstrated in Section 5, EKF and UKF exhibit comparable performance when initial state estimates are close to the true target state. However, under poor initialization and strong nonlinearities, particularly in velocity and angle measurements, UKF consistently outperforms EKF in terms of convergence speed, stability, and robustness. These findings reinforce the suitability of UKF for practical FMCW radar tracking applications involving fast-moving targets.

Several assumptions and limitations of the proposed framework should also be discussed. First, it is assumed that the target range and velocity remain approximately constant over two consecutive chirps due to the short chirp duration. While this assumption holds for fast update rates, its validity may be reduced for longer chirp durations. Second, the proposed method mitigates Doppler ambiguity arising from chirp-to-chirp processing. However, the maximum measurable velocity remains constrained by IF bandwidth, sampling rate, and signal observability conditions. Therefore, lower velocities can be measured at longer distances, while higher velocities can be measured at closer distances. Third, it should be noted that in this study, isolated, single-target tracking was performed in a homogeneous noisy environment. Furthermore, multipath effects were not included in the simulation. Fourth, because the antenna beam is not electronically or mechanically steered in the current design, reliable range and velocity measurements cannot be obtained for targets located at wide angles outside the antenna field of view. Beam steering techniques could be incorporated to address this limitation. Fifth, RF components in the Simulink model are assumed to operate linearly. In practical implementations, nonlinear effects may introduce additional disturbances. Finally, target motion is modeled using a constant-acceleration assumption. For more complex maneuvers, such as rotational or highly agile targets, advanced tracking frameworks such as interacting multiple model (IMM) filters could be considered.

## 7. Conclusions

This paper presented an integrated FMCW radar processing framework for the estimation and tracking of range, velocity, and spatial angle of high-velocity targets at short and medium ranges. A hybrid chirp waveform with bandwidth coding was proposed, enabling accurate velocity estimation using only two chirps while improving the maximum unambiguous velocity limitation inherent in conventional 2D-FFT-based approaches. Simulation results demonstrated that the proposed method achieves reliable velocity measurements for extremely high-speed targets with low IF bandwidth and ADC sampling requirements.

In addition, an amplitude-comparison monopulse antenna configuration was employed for single-snapshot angle estimation, and its performance was evaluated. The integration of nonlinear Kalman filtering further improved tracking accuracy, with the UKF consistently outperforming the EKF in terms of convergence speed and robustness, particularly under poor initial conditions.

Overall, the results suggest that the proposed waveform design and processing framework, within some constraints, supports the detectability and trackability of a high-speed isolated single target by an FMCW radar in a homogeneous environment.

## Figures and Tables

**Table 1:** The parameters of the designed antenna.

Parameter	Value
Element Type	Microstrip Patch Antenna
Element Spacing	$\lambda_{c}/2≅13.65$ mm
Subarray Spacing	$3\lambda_{c}≅41$ mm
Element Taper	Normalized taylorwin(12, 5, −30) from MATLAB
Center Frequency	11 GHz
3 dB Bandwidth	$≅10.8^{{}^{\circ}}$
Operational Bandwidth	500 MHz

**Table 2:** Simulation parameters of FMCW radar front-end.

Parameter	Value
Operating Frequency	11 GHz
Chirp Duration	2 ms
Transmit Power	50 dBm
Noise Floor	−104.9 dBm
Noise Figure	5.6 dB
Maximum IF Bandwidth	2.5 MHz
Sampling Rate	10 Msps
FFT Size	32,768
Antenna Spacing in Subarray	$\lambda_{c}/$2
Subarray Antenna Spacing	$3\lambda_{c}$
Field of View	−6° to 6°

**Table 3:** Actual and estimated results ($B_{1}=B_{2}=$ 300 MHz).

Chirp No.	Range (m)		Velocity (m/s)		Azimuth Angle (Degree)		Elevation Angle (Degree)	
	Actual	Estimated	Actual	Estimated	Actual	Estimated	Actual	Estimated
1	1173	1124	−600.6	*	0	0	0	0
2	675.40	716.60	562.35	*	2.88	2.90	−1.95	−1.90

* No measurement.

**Table 4:** Actual and estimated results ($B_{1}=$ 200 MHz, $B_{2}=$ 300 MHz).

Chirp No.	Range (m)		Velocity (m/s)		Azimuth Angle (Degree)		Elevation Angle (Degree)	
	Actual	Estimated	Actual	Estimated	Actual	Estimated	Actual	Estimated
1	1173.0	1171.7	−600.6	−611.7	0	0	0	0
2	675.4	674.8	−830.0	−864.4	0	0.1	0	0
3	569.3	569.5	−836.6	−861.4	3.59	3.60	1.60	1.60
4	973.8	971.4	−1137.5	−1190.7	2.05	2	0.94	0.95
5	1525.5	1533.5	−1420.6	−1502.3	2.7	2.65	−1.81	−1.85
6	796.7	796.5	−492.0	−507.7	3.98	4	−3.40	−3.40
7	23.0	22.0	−627.7	−657.5	0	0	0	0
8	12.1	11.9	−1472.8	−1556.4	1.65	1.75	−3.30	−3.40
9	276.4	276.8	−924.8	−965.5	−3.88	−3.9	0	0
10	750.4	751.7	−2653.8	−2784.2	1.95	1.90	3.60	3.64
11	967.5	*	−738.0	*	7.74	7.30	1.17	1.20
12	1996.3	1982.8	−6784	−6945.7	0.72	0.7	−1.15	−1.2
13	1996.3	−5615.2	−7230	−65238	*	*	*	*

* No measurement.

**Table 5:** Initial state parameters of the target.

Parameter	Value
Initial Position	(1250, −65, 53) m
Initial Velocity	(−435, 35, 40) m/s
Acceleration	(30, 15, 25) m/s^2^

**Table 6:** Initial filter states at scenario 1.

Parameter	Value
Position	1254.3 m
Velocity	−461.9 m/s
Acceleration	No measurement (0 m/s^2^)
$\mathrm{Initial}\;\theta_{A}$	−3°
$\mathrm{Initial}\;\theta_{E}$	2.4°

**Table 7:** Initial filter states at scenario 2.

Parameter	Value
Position	0 m
Velocity	0 m/s
Acceleration	No measurement (0 m/s^2^)
$\mathrm{Initial}\;\theta_{A}$	−3°
$\mathrm{Initial}\;\theta_{E}$	2.4°

**Table 8:** Comparison of FMCW radar-based velocity estimation methods in the literature.

Ref.	Algorithm	Target	Frequency (GHz)	Waveform	Chirp Number	Chirp Duration	Velocity Error (%)	Max. Velocity (m/s)
[33]	2D FFT	Slow target	10.6	Sawtooth	128	1 ms	8	14
[34]	2D FFT	Slow target	24	Triangular	512	1 ms	1.5	6.25
[3]	2D FFT	Slow target	77	Triangular	3	24 ms	1.6	55.5
[36]	2D FFT	Fast target	20	Sawtooth	-	1.7 μs	0.75	1800
[35]	2D FFT	Slow target	77	Sawtooth	128	50 μs	1	19.5
Ours	Proposed Method	Fast target	11	Bandwidth-coded Sawtooth	2	2 ms	4.7	≅7000 Target at 2000 m

## Data Availability

The original contributions presented in this study are included in the article. Further inquiries can be directed to the corresponding author.
